# Integrating Yin-Yang Concepts in Tai Chi for Geriatric Health: A Holistic Approach to Mind-Body Harmony

**DOI:** 10.1093/geroni/igaf122.1828

**Published:** 2025-12-31

**Authors:** Hao “Howe” Liu

**Affiliations:** Louisiana State University, Baton Rouge, Louisiana, United States

## Abstract

This presentation will focus on applying yin-yang theory in Tai Chi to improve the health and well-being of older adults. It will emphasize the balance between stability and mobility, energy flow, and mental coordination—key elements in supporting aging populations. Tai Chi’s dynamic balance offers a low-impact exercise that can enhance functional independence, circulation, and cognitive health for older individuals.

